# Data on association of the variation (rs1344706) in the ZNF804A gene with schizophrenia and its symptoms in the Russian population

**DOI:** 10.1016/j.dib.2019.103985

**Published:** 2019-05-09

**Authors:** T.V. Lezheiko, D.V. Romanov, N.Yu. Kolesina, V.E. Golimbet

**Affiliations:** a Mental Health Research Center, Moscow, Russia; b I.M. Sechenov First Moscow State Medical University, Russia

**Keywords:** ZNF804A, Risk, Schizophrenia, PANSS, Negative symptoms

## Abstract

The polymorphism rs1344706 in the *ZNF804A* gene is one of the best-supported risk variants for schizophrenia. The association between *ZNF804A* rs1344706 and the disease was demonstrated in many studies but only few of them investigated large samples (above 2000 patients and controls). Data presented show the genotypic distribution of *ZNF804A* rs1344706 in 1265 patients with schizophrenia and 1051 healthy controls from the Russian population. Statistical analysis confirmed the association between rs1344706 and schizophrenia (p = 0.034). The frequency of the risk genotype *AA* was significantly higher in the group of patients compared to that in controls. In addition, the article provides the data on the severity of schizophrenia symptoms measured with the Positive and Negative Syndrome scale (PANSS) in 951 patients. The severity of symptoms was significantly higher in the carriers of the risk genotype *AA* compared to the *AC* genotype and the *CC* genotype.

**Table:** Specifications table

Subject area	**Genetics**
More specific subject area	Psychiatric genetics, association study
Type of data	Table
How data was acquired	Genotyping of DNA samples using PCR System (Applied Biosystems (ThermoFisher) USA), psychometric assessment of symptoms of schizophrenia
Data format	Raw, analyzed.
Experimental factors	Clinical characteristics of patients diagnosed with schizophrenia. Genomic DNA from whole blood samples
Experimental features	Genotyping was performed using a PCR-RFLP assay. Schizophrenia symptoms were assessed with the PANSS
Data source location	Mental Health Research Center, Moscow, Russia
Data accessibility	The data is available within this article
Related research article	Zhu M., Liu T., Zhang J. et al. Association between rs1344706 of ZNF804A and schizophrenia: a meta-analysis Genomics Proteomics Bioinformatics. 12 (2014). Pp. 292–296. https://doi.org/10.1016/j.gpb.2014.10.005

**Table:** 

**Value of the data** • The data can be used in meta-analyses of clinical and molecular-genetic studies of schizophrenia. • Allele and genotype frequencies for ZNF804A rs1344706 in the Russian population can help in population genetic studies. • The data on sex-differences in clinical characteristics of schizophrenia are valuable for epidemiological studies of schizophrenia. • Replication of the association between ZNF804A rs1344706 and schizophrenia in the large samples of patients and controls confirmed the contribution of the AA genotype to the risk of schizophrenia. • The data on the impact of ZNF804A rs1344706 variants on the severity of symptoms of schizophrenia are important for prediction of disease outcomes.

## 1 Data

The dataset reports the frequencies of alleles and genotypes of *ZNF804A* rs1344706 determined in patients with schizophrenia and healthy people from the Russian population (Table 1). Table 2 describes demographic characteristics in carriers of different *ZNF804A* rs1344706 genotypes. Table 3 contains the data on clinical characteristics (age at disease onset and symptom scores) stratified by *ZNF804A* rs1344706 genotype. Table 4 shows the clinical characteristics of male and female patients with different *ZNF804A* rs1344706 genotypes. The distribution of genotypes is different for patients and controls (Chi-square = 6.71, df = 2, p = 0.034). The frequency of the risk genotype *AA* is higher in the group of patients compared to that in controls (p = 0.01). This finding is consistent with other studies [1], [2]. There are no between-genotype differences in age and sex in both groups. ANOVA reveals the effect of sex on the age at disease onset and scores on PANSS positive, negative, general psychopathology subscales (p < 0.05) but not on the total PANSS score. Male patients have younger age at disease onset, lower positive symptom score and higher scores on negative and general psychopathological subscales. The main effect of the *ZNF804A* rs1344706 genotype on the scores on PANSS subscales and the total PANSS score is shown (p < 0.05). The severity of symptoms is higher in the carriers of the risk genotype *AA* compared to the *AC* genotype and the *CC* genotype. Results from other studies support this finding [3], [4].

**Table 1:** Allele and genotype frequencies of *ZNF804A* rs1344706 in patients with schizophrenia and healthy controls.

Alleles and genotypes	Patients (1265)	Controls (1051)
	frequencies of alleles and genotypes	
Allele *А*, %	64	60
Allele *С*, %	36	40
Genotype *АА*, %, (number of patients)	40.79 (516)	36.82 (387)
Genotype *АС*, %, (number of patients)	46.17 (584)	46.81 (492)
Genotype *СС*, %, (number of patients)	13.04 (165)	16.37 (172)

**Table 2:** Demographic characteristics of the subjects with different *ZNF804A* rs1344706 genotypes.

Controls				
Variables	*AA* (n = 387)	*AC* (n = 492)	*CC* (n = 172)	Total (n = 1051)
Men, n (%)	206 (36.9)	256 (46.0)	95 (17.1)	557 (53)
Age, years, mean (SD)	29.3 (10.6)	29.1 (9.6)	29.1 (10.7)	29.2 (10.1)
Women, n (%)	181 (36.6)	236 (47.8)	77 (15.6)	494 (47)
Age, years mean (SD)	30.1 (11.2)	32.2 (11.9)	35.3 (16.2)	31.9 (12.5)
Patients				
Variables	*AA* (n = 516)	*AC* (n = 584)	*CC* (n = 165)	Total (n = 1265)
Men, n (%)	222 (40.4)	258 (46.9)	70 (12.7)	550 (43.5)
Age, years mean (SD)	30.3 (12.1)	31.5 (11.8)	28.7 (9.6)	30.8 (11.7)
Women, n (%)	294 (41.1)	326 (45.6)	95 (13.3)	715 (56.5)
Age, years mean (SD)	38.2 (13.3)	37.3 (12.2)	39.6 (12.9)	38.1 (13.2)

**Table 3:** Clinical characteristics of patients with schizophrenia by ZNF804A rs1344706 genotype.

ZNF804A rs1344706 genotype				
Variables, mean (SD)	*AA* (n = 389)	*AC* (n = 430)	*CC* (n = 132)	Total (n = 951)
Age at onset, years	23.8 (9.8)	24.0 (9.7)	23.6 (8.7)	23.8 (9.6)
PANSS (positive symptoms), score	24.1 (8.4)	24.1 (9.0)	21.7 (7.8)	23.7 (8.6)
PANSS (negative symptoms), score	23.1 (7.9)	21.3 (7.2)	21.7 (7.1)	22.1 (7.5)
PANSS (general psychopathological symptoms), score	40.3 (13.1)	37.9 (12.9)	37.8 (13.2)	38.9 (13.1)
PANSS (total) score	87.0 (21.9)	82.9 (20.5)	81.3 (22.1)	84.4 (21.4)

**Table 4:** Clinical characteristics of male and female patients with different ZNF804A rs1344706 genotypes.

Male patients with schizophrenia				
*ZNF804A* rs1344706 genotype				
Variables, mean (SD)	*AA* (n = 133)	*AC* (n = 135)	*CC* (n = 46)	Total (n = 314)
Age at onset, years	21.6 (8.6)	20.6 (6.1)	21.5 (5.7)	21.2 (7.3)
PANSS (positive symptoms), score	22.1 (7.6)	21.5 (7.3)	19.1 (7.0)	21.4 (7.4)
PANSS (negative symptoms), score	24.5 (6.6)	23.0 (6.5)	20.6 (5.2)	23.2 (6.4)
PANSS (general psychopathological symptoms), score	42.8 (10.6)	39.6 (11.2)	38.2 (10.7)	40.6 (11.0)
PANSS (total), score	87.8 (18.4)	83.1 (19.2)	78.0 (18.5)	84.4 (19.0)

Female patients with schizophrenia				
*ZNF804A* rs1344706 genotype				
Variables, mean (SD)	*AA* (n = 256)	*AC* (n = 295)	*CC* (n = 86)	Total (n = 637)
Age at onset, years	25.9 (10.2)	26.7 (10.6)	25.4 (10.1)	26.3 (10.3)
PANSS (positive symptoms), score	24.5 (8.6)	25.3 (9.4)	23.1 (7.9)	24.8 (8.9)
PANSS (negative symptoms), score	22.5 (8.3)	20.5 (7.4)	22.3 (7.9)	23.2 (6.5)
PANSS (general psychopathological symptoms), score	39.2 (13.6)	37.1 (13.6)	37.6 (14.4)	38.0 (13.9)
PANSS (total), score	86.6 (23.5)	82.7 (21.0)	83.1 (23.6)	84.5 (19.0)

## 2 Experimental design, materials and methods

### 2.1 Sample collection and genomic DNA extraction

Schizophrenia sample was selected from patients admitted to psychiatric units of the Mental Health Research Center. The sample included 1265 people, 550 men, 715 women, mean age 34.8 (13.7) years. Healthy controls without a family history of mental diseases were enrolled from Moscow and Moscow region. The control group included 1051 people, 557 men, 494 women, mean age 30.5 (11.4) years. All participants were ethnically Russian. All of them provided the written informed consent for participation in the study. DNA from whole blood was extracted using the standard phenol-chloroform method.

### 2.2 DNA genotyping

Genotyping was performed using a PCR-RFLP assay [5]. The following primers were used: forward, 5′-AGTGACCTTGGTGGAAATGG-3′and reverse, 5′-TTTTCCAGGTAGGGGATTGG-3. The amplified products were separated in the 8% polyacrylamide gel, stained and visualized under UV light. Genotype frequencies did not deviate from the Hardy- Weinberg equilibrium in both patients (Chi2 = 0; p < 0.05) and control subjects (Chi2 = 0,55; p < 0.05).

### 2.3 Phenotyping

The diagnosis of schizophrenia was made according to criteria of The International classification of Diseases 10th revision (ICD-10). Clinical symptoms were measured using the Positive and Negative Syndromes Scale (PANSS) [6], a widespread instrument proven to be valid and suitable for evaluation of positive, negative and general psychopathological items. The PANSS interviews were conducted one week before the patient's discharge from the hospital. Because sex-modulated association of *ZNF804A* with schizophrenia [7], [8] was reported in earlier studies, we additionally considered clinical characteristics separately in men and women.

### 2.4 Statistical genetic analyses

Allele and genotype frequencies in cases and controls were compared using 2 chi-square contingency tables. ANCOVA with Bonferroni post-hoc test was used with PANSS score or age at onset as a dependent variable and genotype (*AA* vs *AC* vs *CC*) and sex as between-subject factors with age as a covariate.
