# Supplementary material for: Data on association of the variation (rs1344706) in the ZNF804A gene with schizophrenia and its symptoms in the Russian population
Source: Data Brief. 2019 May 9;24:103985. doi: 10.1016/j.dib.2019.103985 (PMC6536608; doi:10.1016/j.dib.2019.103985)
Supplement: Multimedia component 1 [file mmc1.doc]

13st March, 2019

The Editor,

Data In Brief,

Dear Sir,

**DECLARATION OF CONFLICT OF INTEREST**

I, Dr. Vera Golimbet and my colleagues declare that there is no conflict of interest traceable to our data paper “***Data on association of the variation (rs1344706) in the ZNF804A gene with schizophrenia and its symptoms in the Russian population***”

Vera Golimbet
